# Epitope Capsid-Incorporation: A New Effective Approach for Vaccine Development for Chagas Disease

**DOI:** 10.20411/pai.v1i2.114

**Published:** 2016-09-15

**Authors:** Qiana L. Matthews, Anitra L. Farrow, Girish Rachakonda, Linlin Gu, Pius Nde, Alexandre Krendelchtchikov, Siddharth Pratap, Shruti S. Sakhare, Steffanie Sabbaj, Maria F. Lima, Fernando Villalta

**Affiliations:** 1 Department of Biological Sciences, Alabama State University, Montgomery, Alabama; 2 Department of Medicine, Division of Infectious Diseases, University of Alabama at Birmingham, Alabama; 3 Department of Microbiology and Immunology, School of Medicine, Meharry Medical College, Nashville, Tennessee; 4 Division of Pulmonary, Allergy and Critical Medicine, Department of Medicine, University of Alabama at Birmingham, Birmingham, Alabama

**Keywords:** Chagas disease, *T. cruzi* vaccine constructs, epitope-capsid incorporation, trypomastigote gp83 neutralizing epitope, amastigote surface protein 2 epitope, co-immunization, immuno-protection

## Abstract

**Background:**

Previously we reported that a hexon-modified adenovirus (Ad) vector containing the invasive neutralizing epitope of *Trypanosoma cruzi* (*T. cruzi)* trypomastigote gp83 (Ad5-gp83) provided immunoprotection against *T. cruzi* infection. The purpose of this work was to design an improved vaccine for *T. cruzi* using a novel epitope capsid incorporation strategy. Thus, we evaluated the immunoprotection raised by co-immunization with Ad5-gp83 and an Ad vector containing an epitope (ASP-M) of the *T. cruzi* amastigote surface protein 2.

**Methods:**

Protein IX (pIX)-modified Ad vector (Ad5-pIX-ASP-M) was generated, characterized, and validated. C3H/He mice were immunized with Ad5-pIX-ASP-M and Ad5-gp83 and the cell-mediated responses were evaluated by enzyme-linked immunospot (ELISPOT) assay and intracellular staining. Immunized mice were challenged with *T. cruzi* to evaluate the vaccine efficacy.

**Results:**

Our findings indicate that Ad5-pIX-ASP-M was viable. Specific CD8^+^ T-cell mediated responses prior to the challenge show an increase in IFNγ and TNFα production. A single immunization with Ad5-pIX-ASP-M provided protection from *T. cruzi* infection, but co-immunizations with Ad5-pIX-ASP-M and Ad5-gp83 provided a higher immunoprotection and increased survival rate of mice.

**Conclusions:**

Overall, these results suggest that the combination of gp83 and ASP-M specific epitopes onto the capsid-incorporated adenoviruses would provide superior protection against Chagas disease as compared with Ad5-gp83 alone.

## INTRODUCTION

Chagas disease is a neglected disease that affects 8–15 million people in Latin America. The disease has now spread globally due to international human migration, and it is becoming a new worldwide health challenge [1, 2]. Currently, 2–7 million people with Chagas disease live in North America [3]. Chagas-infected individuals represent a $7 billion/year burden worldwide [4]. The existing drugs are toxic and have limited efficacy and recent clinical trials with new drugs (posaconazole and ravuconazole) failed [5, 6]. Rational drug discovery based on the structure of drug targets for *Trypanosoma cruzi* (*T. cruzi)* has yielded two promising drugs (VNI and VFV), which have not yet entered clinical trials [7–10].

Innate and adaptive immunity play important roles in parasite growth control during the acute infection; however, the parasite suppresses the immune system, allowing the establishment of the destructive chronic phase. To date, no preventive or therapeutic human vaccines have entered clinical trials. Thus, there is a desperate need for a safe and effective vaccine to protect the 40–100 million individuals at risk. Defined molecular vaccines for Chagas disease would be ideal to overcome controversial potential molecular mimicry and immunosuppression caused by the parasite [9–17].

Efforts to generate experimental vaccines using Chagas animal models with inactivated and attenuated parasites [18], purified proteins, recombinant proteins DNA, and, more recently, replication-deficient bacteria and recombinant vectors were reported [19–21]. However, progress made with defined molecular vaccines is minimal.

Recently, we reported a novel strategy for generating a new effective vaccine for Chagas disease consisting of incorporating a *T. cruzi* epitope (gp83) into the capsid of modified Adenovirus-5 (Ad5) [22]. We found that mice immunized with this construct provided protection against *T. cruzi* by reducing infection, inducing neutralizing antibodies, and increasing survival rates [22]. The surface glycoprotein 83 (gp83) is a trans-sialidase like molecule unique to invasive trypomastigotes and used as a ligand to attach to host cells and initiate infection [23]. Blocking gp83 with MAb 4A4, which recognizes a gp83 epitope, neutralizes trypomastigote cellular infection [24]. Passive immunization with monovalent 4A4 Fab fragments neutralizes *T. cruzi* infection in mice challenged with a lethal dose of trypomastigotes [23]. In efforts to continue the development of the most effective vaccine for *T. cruzi*, we extended our antigen capsid-incorporation strategy to include both a humoral and cellular response. To date, one of the most promising candidates for a Chagas disease vaccine has been the amastigote protein 2 (ASP-2) [25–29]. We reasoned that a vaccine including the gp83 neutralizing epitope and an epitope (ASP-M) of ASP-2 would provide additional significant protection by inducing neutralizing antibodies, beneficial CD8^+^ cellular responses, reduction of parasitism, and increased survival rate. Here, we provide evidence that the ASP-M epitope was incorporated into the capsid protein pIX of Ad5. We also confirmed that upon challenge with a lethal dose of trypomastigotes, the mice co-immunized with the gp83 neutralizing epitope, and the ASP-M epitope capsid-incorporated vector displayed a significant reduction in parasitemia, improvement of their survival rate by eliciting neutralizing antibodies and CD8^+^ T cells capable of stimulating CD107a, TNFα, and IFNγ in response to the ASP-M epitope.

## METHODS

### Cell Culture and Parasites

Human embryonic kidney (HEK293) cells were obtained from and cultured in the medium recommended by the American Type Culture Collection (Manassas, VA).

*T. cruzi* Tulahuen blood trypomastigotes [30] were used for challenging immunized mice [7]. Trypomastigotes expressing green fluorescent protein (GFP) for cellular infection assays were generated as described [31].

### Recombinant Adenoviral Construction

Recombinant adenovirus with the *T. cruzi* ASP-M epitope as well as His_6_ genetically incorporated within Ad5 pIX was generated [32]. Briefly, the DNA sequence corresponding to the median immunodominant region of ASP-2 and His_6_ (24 amino acids) was generated by GenScript (Piscataway, NJ) and subcloned into the pIX shuttle vector to generate pIX-shuttle-ASP-M. The resulting plasmid was then digested with PmeI. The digested fragment containing the homologous recombination regions and the pIX gene were recombined through homologous recombination with an Ad5 backbone replacing the wildtype pIX gene. The recombination was performed in *Escherichia coli* BJ5183, leading to the identification of positive vector clones.

### Rescue, Purification, and Titration of Recombinant Ad5 Vector

To rescue the vector, the recombinant adenoviral genome was digested with PacI and transfected with PolyJet (SignaGen Laboratories) into the Ad5-E1-expressing HEK293 cells. Multi-step large-scale propagations of recombinant Ad5 vector were performed after the vector was rescued. Viruses were purified by double CsCl ultracentrifugation. Physical titers, expressed as viral particles (VPs) per mL, were measured at OD 260 nm. Infectious particles (IPs) per mL were determined by tissue culture infectious dose (TCID_50_) assay [33].

To confirm the ASP-M epitope-His_6_ incorporation on the hexon gene, PCR analysis was performed with the following primers: 5′-CAATTGGATTCTTTGACCC-3′ and 5′ AATTTGTC CCGTCTCCCATTCGGT-3′.

### Western Blot Analysis

To analyze the ASP-M epitope and His_6_ expression, immunoblots of 5x10^9^ VPs/vector were probed with His_6_ MAb and developed with HRP-conjugated goat anti-mouse antibody. Proteins were detected by 3′3′-diaminobenzidine [33].

### Whole Virus ELISAs

To investigate the exposure-display of ASP-M epitope and His_6_ on the surface of the capsid, whole virus enzyme-linked immunosorbent assays (ELISAs) were performed [34]. Different amounts of the Ad5-pIX-ASP-M or Ad5 (control) were immobilized onto 96-well plates, incubated with His_6_ MAb, HRP-conjugated goat anti-mouse antibody, developed with peroxidase substrate and measured at OD 450 nm.

### Mice Immunizations

C3H/He mice (6 weeks) were immunized with either Ad5 (control) or Ad5-pIX-ASP-M to determine the ASP-M-specific immunogenicity using IACUC approved protocols. Mice groups were immunized intramuscularly with the corresponding vector (1x10^10^ VP/mouse) at each time-point, with a two-week interval between prime, boost, and reboost.

### IFNγ ELISPOT Assays

For IFNγ enzyme-linked immunospot (ELISPOT) assays, groups of mice were immunized as described previously. Two weeks after boost immunization, peripheral blood mononuclear cells (PBMCs) were collected. All assays were carried out using Mouse IFNγ ELISPOT Ready-Set-Go Kit (eBioscience, San Diego, CA). Nitrocellulose plates were coated with 10 μg/mL of IFNγ capture antibody. PBMCs were seeded at 2 × 10^5^ cells/well in triplicate and stimulated with ASP-M peptide (10 μg/mL) or media alone. For positive control, PBMCs were stimulated with 10 ng/mL phorbol 12- myristate 13-acetate (PMA) plus 500 ng/mL ionomycin. After incubation, cells were removed and spot forming cells (SFCs) were enumerated using CTL ImmunoSpot S6 Ultra V Analyzer.

### Intracellular Cytokine Staining Assay

Splenocytes from C3H/He immunized mice were treated with ACK lysing buffer (Thermo Fisher Scientific, Waltham, MA) and the cell concentration was adjusted to 2 × 10^6^ cells/mL in 500 μl of cell culture medium containing CD107a-FITC (2 mg/mL), and Golgi stop (monensin) [10μg/mL], (BD Biosciences, San Jose, CA). The ASP-M peptide, TEWETGQI (10μM) was added to the experimental tubes; PMA (50 ng/mL) and ionomycin (1μg/mL) were added to the positive control tubes and incubated for 6 hours. Cells were incubated with surface antibodies and stained with CD3-Pacific Blue hamster-anti-mouse (BD Biosciences, San Jose, CA), CD4-APC-eFluor 780 anti-mouse (eBioscience, San Diego, CA), and CD8-PE rat-anti-mouse (BD Biosciences, CA) to determine the surface phenotype. Cells were permeabilized with Cytofix/Cytoperm (BD Biosciences, CA), stained for intracellular markers TNFα-PE-Cy7 rat-anti-mouse and IFNγ-Alexa Fluor 700-rat-anti-mouse (BD Biosciences, CA), and fixed in 1% formalin. At least 100 000 CD3^+^ events were acquired from each sample using a Becton Dickinson LSR II flow cytometer (BD Biosciences, CA) and data was analyzed using FlowJo v10 software (Tree Star, Ashland, OR). Lymphocytes were analyzed based on forward and side scatter profiles. Gates were set based on the media control and applied to all samples from the same individual for each time point. Cytokines produced were measured from the CD3^+^CD4^+^ or the CD3^+^CD8^+^ gates relative to the media control values.

### Mice Challenge

Groups of 5 female C3H/He mice (Jackson Laboratory, 6-week-old, same weight) immunized with Ad5 (control), Ad5-pIX-ASP-M, Ad5-gp83 or with Ad5-gp83 + Ad5-pIX-ASP-M (as described previously) were challenged intraperitoneally with a lethal dose of 5 × 10^3^ blood trypomastigotes of the clone 20A of the Tulahuen strain of *T. cruzi* using Institutional Animal Care and Use Committee-approved protocols. Parasitemia was monitored in 5 μl of mouse tail blood [18]. The survival rate was recorded.

### Neutralization Assay

Mice immunized with Ad5-pIX-ASP-M, Ad5-gp83, Ad5-gp83 + Ad5-pIX-ASP-M or nonimmunized control mice were bled before *T. cruzi* challenge to obtain serum to evaluate the ability of antibodies to neutralize *T. cruzi* infection of cardiomyocytes [22]. GFP-expressing trypomastigotes were pre-incubated with sera from immunized or nonimmunized control mice for 30 minutes at 37°C and exposed in triplicate to cardiomyocyte monolayers at 10 parasites/cell ratio [12]. Parasite multiplication within cell monolayers at 72 hours was determined fluorometrically as relative fluorescence units (RFU) [31, 35]. To microscopically visualize the effect of neutralizing antibodies on cellular infection, we repeated the aforementioned experimental conditions. Cells were fixed and stained with 4′, 6-diamidino-2-phenylindole (DAPI) and Alexa Fluor 546 phalloidin for fluorescence confocal microscopy evaluation of infection [7, 35].

## STATISTICAL ANALYSES

Statistical analyses were performed by the nonpaired 2-tailed Student's t-test, assuming equal variance and analysis of variance (ANOVA) when appropriate. Statistical significance was defined as *P* < 0.05.

## RESULTS

### Construction and Characterization of Ad5 Vector with Modified Protein IX

**Figure 1.**
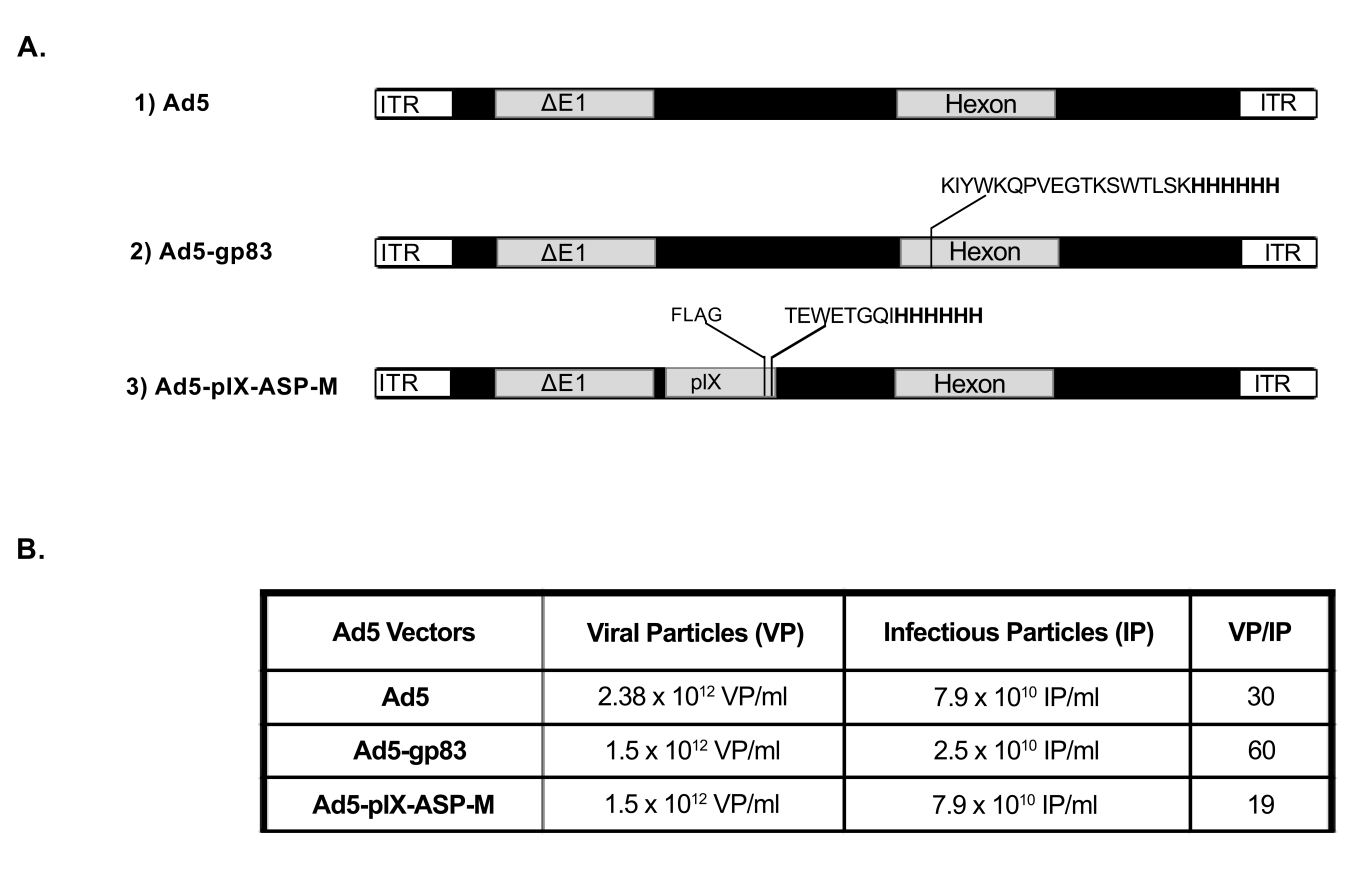
**Schematic representation of the *T. cruzi* ASP-M epitope and His_6_ epitope genetically incorporated into the pIX of Ad5 and *T. cruzi* gp83 epitope incorporated into the hexon of Ad5 (A), and viro-logical properties of vectors (B).** (A): (1) Ad5, a replication-defective adenovirus with wildtype pIX. (2) Ad5-gp83, Ad5 replication-defective genome containing an incorporated neutralizing *T. cruzi* trypomastigote gp83 epitope within the hexon locale. (3): Ad5-ASP-M, Ad5 replication-defective genome containing an incorporated *T. cruzi* amastigote ASP-M epitope within the pIX locale. (B): VP/IP ratio.

Our recent antigen capsid-incorporation studies have utilized the Ad5 hexon. In this body of work, we explored the use of antigen capsid-incorporation by modifying Ad5 pIX as a novel tool for vaccine development. The modified Ad vector, referred to as Ad5-pIX-ASP-M (Figure 1A), contains the immunodominant CD8^+^ T-cell epitope (TEWETGQI) from the medial region of ASP-2 [36] as well as His_6_ and Flag (DYKDDDDK) epitopes incorporated into the minor Ad capsid protein IX (pIX) (Figure 1A, construct 3). The control vector (Ad5) and Ad5-gp83 vector depicted in Figure 1A were generated as described [22]. Ad5-pIX-ASP-M was rescued and upscaled as described [33]. The physical and infectious titers were determined to test the stability of the Ad5-pIX-ASP-M vector. Based on those two assays, the viral particle/infectious particle (VP/IP) ratio was determined (Figure 1B). A normal VP/IP ratio of unmodified Ad ranges from ~10–30 [33]. The VP/IP ratios for Ad5 and Ad5-gp83 were 30 and 60, respectively. For Ad5-pIX-ASP-M, the VP/IP ratio was 19. Based on these observations, the insertion of the ASP-FLAG-His_6_ epitope did not affect vector stability.

**Figure 2.**
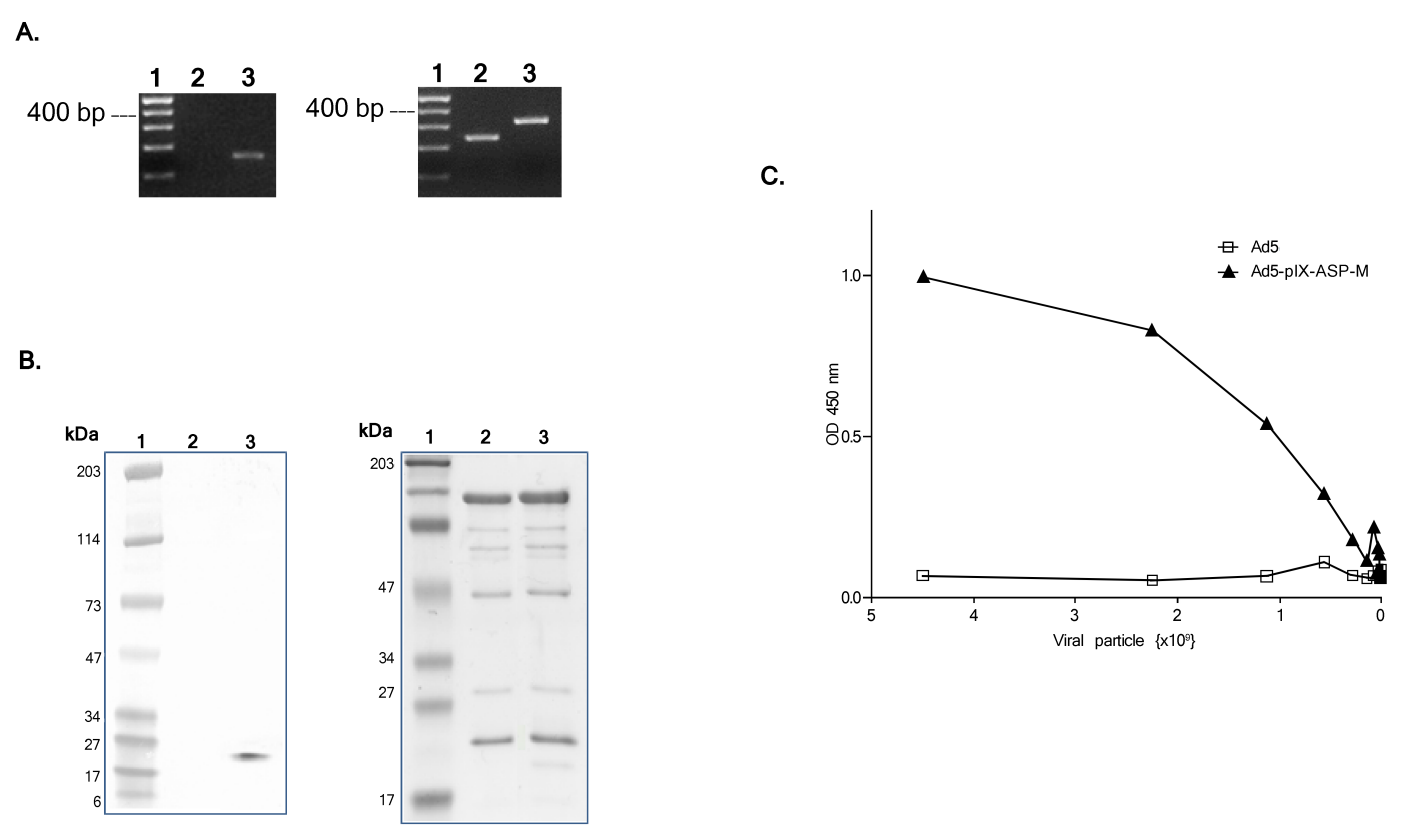
**Verification of epitope capsid-incorporation into pIX of Ad5 (A and B) and epitope exposure on the virion surface (C).** (A): left panel A, pIX-specific PCR primers confirmed the presence of the ASP-M DNA within the pIX locale. Lane 1, DNA ladder; lane 2, Ad5; and lane 3, Ad5-pIX-ASP-M. Right panel A, *T. cruzi*-His_6_-specific primers confirmed the incorporation of ASP-M and His_6_ DNA. Lane 1, DNA ladder; lane 2, Ad5; and lane 3, Ad5-pIX-ASP-M. (B): Left panel B, Immunoblots confirmed the presence of His_6_ incorporation within the modified vector. Protein marker (lane 1), Ad5 (lane 2), and Ad5-pIX-ASP-M (lane 3). Right panel B, Coomassie blue staining of the Ad5 vectors. Protein marker (lane 1), Ad5 (lane 2), and Ad5-pIX-ASP-M (lane 3). (C): Varying amounts (starting at 4.5 × 10^9^ VP/mouse) of Ad5 or Ad5-pIX-ASP-M were immobilized onto the wells of ELISA plates, incubated with His_6_ MAb and HRP-conjugated secondary antibody and the OD was read at 450 nm.

PCR analysis of the vector confirmed the antigen capsid-incorporation. As shown in Figure 2A left panel lane 3, the amplification product of expected size (165 bp) was found for Ad5-pIXASP-M and no amplification in the Ad5 control lane was seen (Figure 2A, left panel, lane 2). For the pIX-specific PCR, Ad5 yielded a 272 bp band (Figure 2A, right panel, lane 2) whereas Ad5-pIX-ASP-M yielded a higher band at 323 bp indicating the incorporation of the ASP-FLAG-His_6_ DNA within the pIX region (Figure 2A, right panel, lane 3).

Ad5-pIX-ASP-M contains a protein band that was detected with Flag HRP antibody as 18.5 kDa (Figure 2B, left panel, lane 3), as expected for the ASP-FLAG-His_6_ incorporation within pIX.

Ad5-pIX-ASP-M displayed a similar protein profile as the control Ad5 vector (Figure 2B, right panel), indicating that the ASP-FLAG-His_6_ incorporation did not affect the expression of any of the Ad capsid proteins.

### Incorporated ASP Antigen Accessible on the Surface of the Virion

ELISA assays confirmed that the *T. cruzi* antigen was virion surface accessible (Figure 2C). This is critical with respect to pIX-modified vectors because Ad vectors can be produced that are pIX defective [37] and in order to generate an appropriate immune response, the pIX-ASP protein must be virion surface exposed. Dose-dependent binding of the Ad5-pIX-ASP-M vector was seen, demonstrating that the *T. cruzi* antigen was capsid-incorporated and surface exposed, whereas no binding was seen in response to Ad5 control.

### Cellular Immune Response of Mice Immunized with the Modified Ad5 Vector

**Figure 3.**
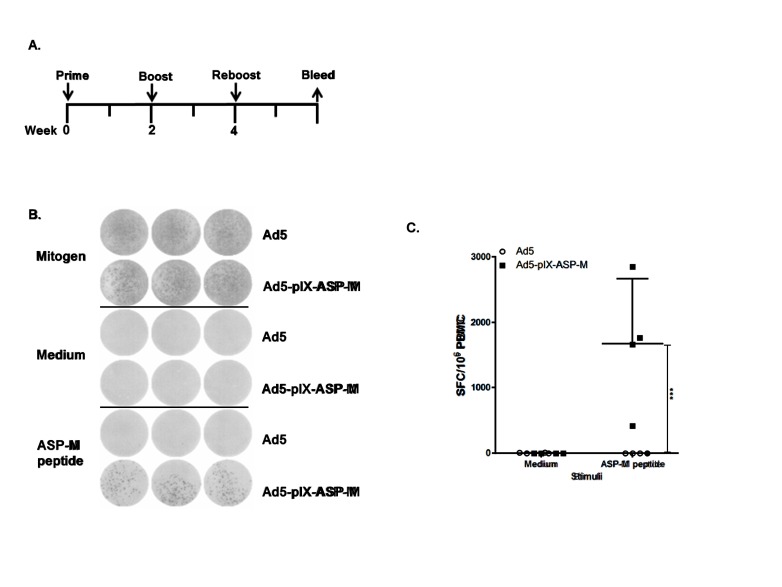
**Immunization with Ad5-pIX-ASP-M induces IFNγ-secreting cells.** (A): Animals were immunized every 2 weeks for a total of 3 immunizations. PBMCs were obtained 2 weeks following the final immunization and were assayed by enzyme-linked immunosorbent spot (ELISPOT). (B): A representative ELISPOT. Well images of 4 × 10^5^ PBMCs cultured with media, mitogen (PMA/ionomycin), or ASP-M peptides. (C): Analysis of ASP-M IFNγ-producing cells in PBMC upon immunization with Ad5-pIX-ASP-M. Each square or dot represents the mean number of IFNγ secreting cells per 10^6^ PBMC for an individual mouse. (***) = *P* < 0.001.

**Figure 4.**
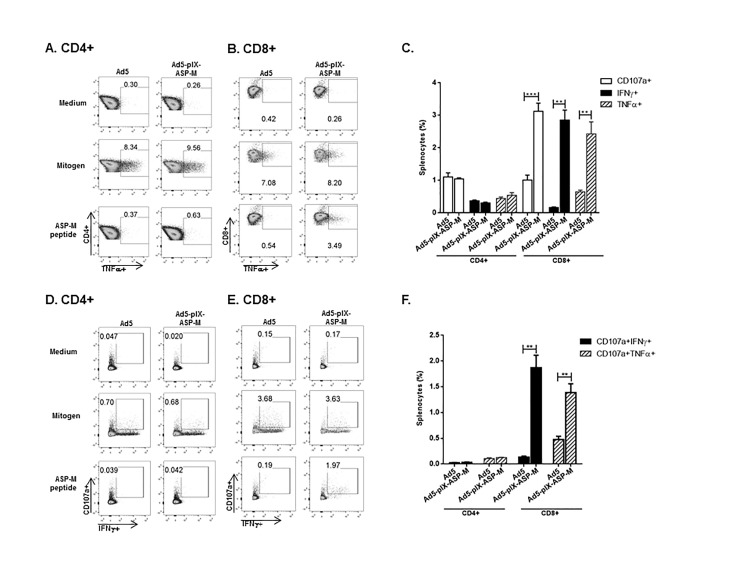
**Frequencies of ASP-specific T-cell responses in immunized mice.** Representative flow cytometric analysis of intracellular staining for IFNγ and TNFα production by ASP-M-specific CD4^+^ and CD^8^+ T-cells memory cells. C3H/He mice were immunized with Ad5 or Ad5-pIX-ASP-M as described in Materials and Methods. Two weeks after the last immunization, splenocytes harvested from a subset of mice were stimulated with anti-CD107a and GolgiStop in the presence or absence of ASP-M peptide. After 6 hours, cells were incubated with anti-CD3, anti-CD4, and anti-CD8. Next the cells were permeabilized and fixed, then stained with anti-TNFα, and anti-IFNγ. Cells were gated by side scatter area (SSC-A); forward scatter area (FSC-A); and gated for CD3^+^ CD4^+^ or CD^3+^ CD^8+^ T lymphocytes. (A): Representative flow cytometry plots of TNFα^+^ by CD4^+^ T cells from the spleens of the immunized mice; (B): Representative flow cytometry plots of TNFα^+^ by CD8^+^ T cells from the spleens of the immunized mice; (C): Histogram showing the percentage of CD107a^+^, INFγ^+^, TNFα^+^ production. The numbers represent the frequencies of cells stained for CD4^+^INF^+^, CD4^+^TNFα^+^ or CD8^+^INF^+^, CD8^+^TNFα^+^. The results are presented as the mean ± SEM frequencies of CD4^+^ or CD8^+^ cells for 4 mice. (**) = *P* < 0.01 and (***) = *P* < 0.001. (D): Representative flow cytometry plots of CD107a^+^IFNγ^+^ produced by CD4^+^ T cells from the spleens of the immunized mice; (E): Representative flow cytometry plots of CD107a^+^IFNγ^+^ produced by CD8^+^ T cells from the spleens of the immunized mice; (F) Histogram showing the percentage of CD107a^+^IFNγ^+^ and CD107a^+^TNFα^+^ production. The results are presented as the mean ± SEM frequencies of CD4^+^ or CD8^+^ cells for 4 mice. (**) = *P* < 0.01.

We assessed whether immunization with Ad5-pIX-ASP-M could elicit a cell-mediated *T. cruzi-*specific immune response. Two weeks after homologous vector reboost immunization, as shown in the immunization schedule (Figure 3A), C3H/He mice PBMCs were evaluated for IFNγ production by ELISPOT following stimulation with mitogen (PMA/ionomycin), or ASP-M peptide, presenting putative binding sites to MHC class I (H-2K^k^) [36] and to HLA-A* 32:05, HLA-A* 32:08, HLA-A* 02:87, HLA-A* 32:06, and HLA-A* 32:20, that we identified using NetMHCpan [37], which are alleles frequent in Hispanic populations (www.allelefrequencies.net), and to 10 additional HLA-A* 02 and 32 alleles. ELISPOT analysis revealed measurable numbers of spot forming cells (SFCs) in the PBMCs stimulated with mitogen and the ASP-M peptide (Figure 3). Mitogen stimulated PBMCs produced greater than 1000 SFCs (Figure 3B). PBMCs from mice immunized with Ad5-pIX-ASP-M and stimulated with TEWETGQI produced an average of 1676 SFCs whereas PBMCs from Ad5 immunized mice produced less than 5 SFCs (Figure 3C). The significant differences in the number of IFNγ producing cells in the mice immunized with Ad5-pIX-ASP-M compared with the mice immunized with Ad5 (*P* < 0.001) allowed us to conclude that Ad5-pIX-ASP-M elicit a strong cell-mediated immune response. Two weeks after homologous vector reboost immunization; splenocytes from a subset of immunized mice were stimulated with mitogen or ASP-M peptide and assessed for intracellular staining of IFNγ and TNFα cytokines and for the surface mobilization of CD107a, a marker of T-cell degranulation. Representative histograms depicting the gating strategy for the detection of TNFα from both CD4^+^ and CD8^+^ T cells are shown in Figure 4A and 4B. There was no difference in the frequencies of CD4^+^ T lymphocytes producing CD107a, IFNγ, and TNFα in response to the ASP-M peptide in the Ad5-pIXASP-M immunized mice compared with the Ad5 immunized mice (Figure 4C), indicating that the ASP-M epitope is MHC class I CD8 restricted. However, we observed substantially higher production of CD107a (*P* < 0.001), IFNγ (*P* < 0.01), and TNFα (*P* < 0.01) secretion in response to the ASP-M peptide from CD8^+^ T-cells isolated from Ad5-pIX-ASP-M immunized mice compared with the CD8^+^ T-cells from Ad5 immunized mice (Figure 4C).

Similar results were observed when CD4^+^ and CD8^+^ T cells were analyzed for dual effector molecule secretion (IFNγ^+^CD107a^+^ and TNFα^+^CD107a^+^). Representative histograms of the double-positive gating for IFNγ^+^CD107a^+^ cells are shown in Figure 4(D and E). There was no significant difference in the percentage of CD4^+^IFNγ^+^CD107a^+^ or CD4^+^TNFα^+^CD107a^+^ cells in Ad5-pIX-ASP-M immunized mice compared with the Ad5 immunized mice (Figure 4F). There was a significant difference in the frequency of CD8^+^ T cells double-positive for IFNγ^+^CD107a^+^ secretion (*P* < 0.01) as well as the frequency of TNFα^+^CD107a^+^ secreting cells (*P* < 0.01) in the immunized mice compared with the control group (Figure 4F). These results demonstrate that the IFNγ detected in our previous ELISPOT assays was secreted by CD8^+^ T cells.

### Modified Ad5 Vectors That Induced Protective Immunity From T. Cruzi Infection

**Figure 5.**
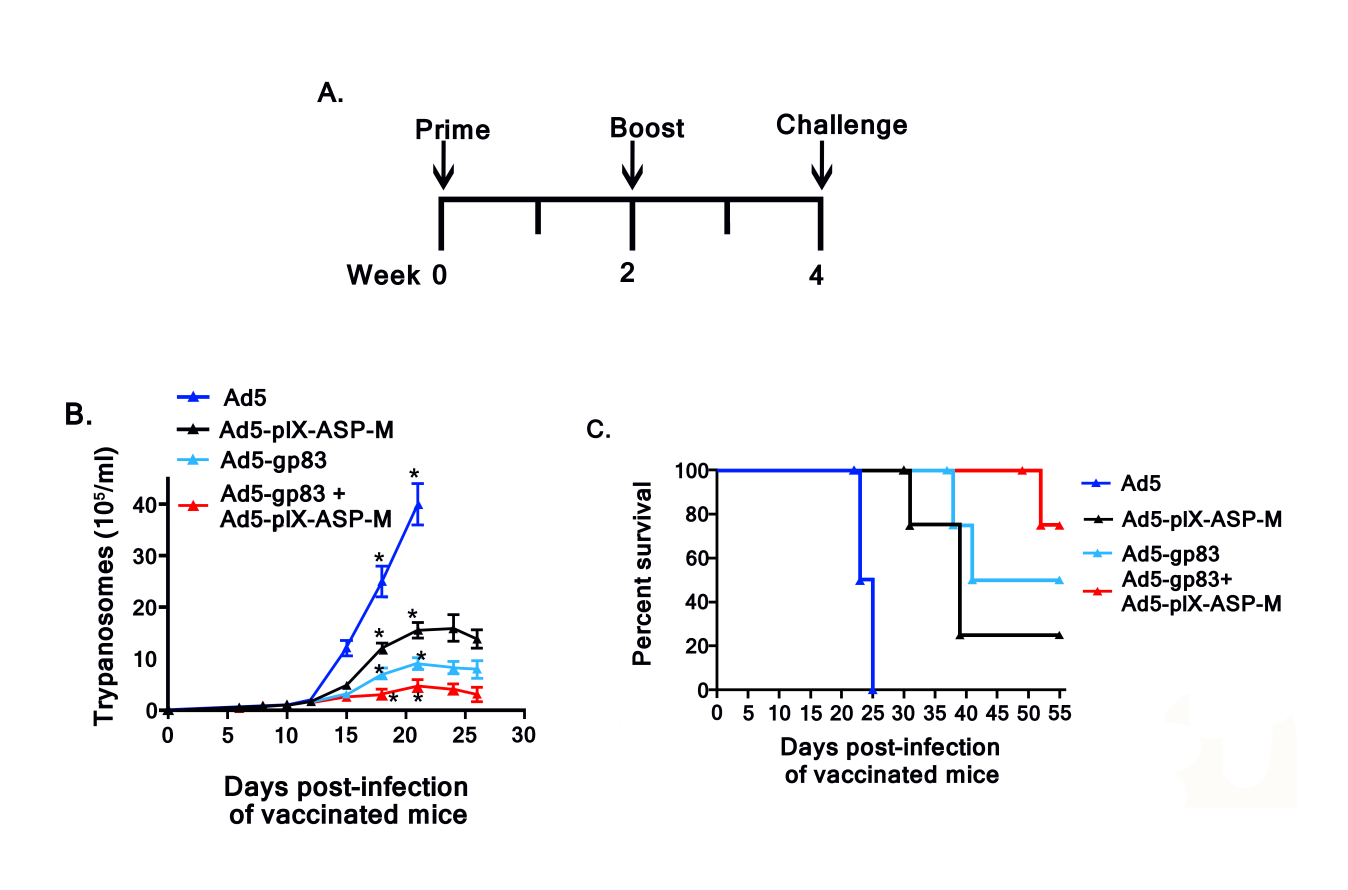
**Immunization of mice with Ad5-pIX-ASP-M provides protection against challenge with *T. cruzi*, and this protection is significantly increased when mice are co-immunized with Ad5-pIXASP-M and Ad5-gp83.** (A): Schedule of immunization and challenge with *T. cruzi* trypomastigotes. (B) Parasitemia of vaccinated mice with several vaccine Ad vector constructs. C3H/He mice (5 per group, 6-week-old) were immunized with Ad5, Ad5-pIX-ASP-M, Ad5-gp83 or with Ad5-gp83 + Ad5-pIXASP-M and challenged intraperitoneally with a lethal dose of blood trypomastigotes (5 × 10^3^). The kinetics of parasitemia was determined in 5 μl of blood tail. Data represent the mean values ± SEM. The means are significantly different (*P* < 0.0229) among the 4 groups at the times indicated by 1-way ANOVA. (C): Kaplan–Meier survival plot.

C3H/He mice were immunized with Ad5, Ad5-pIX-ASP-M, Ad5-gp83 or together with Ad5-gp83 + Ad5-pIX-ASP-M, according to the immunization schedule depicted in Figure 5A. Two weeks after boost, mice were injected with a lethal dose of blood trypomastigotes. Mice immunized with vector Ad5-pIX-ASP-M, Ad5-gp83 or together with Ad5-gp83+ Ad5-pIX-ASP-M and challenged with a lethal dose of *T. cruzi* blood trypomastigotes. Mice immunized with vector Ad5-pIX-ASP-M showed ~60% reduction in parasitemia whereas mice immunized with the Ad5-gp83 vector showed ~70% reduction compared with the mice immunized with Ad5 alone. Interestingly, mice immunized with Ad5-gp83 + Ad5-pIX-ASP-M and challenged with a lethal dose of *T. cruzi* blood trypomastigotes presented the most parasitemia reduction at ~80% with respect to the mice group that received Ad5 vector alone (Figure 5B).

**Figure 6.**
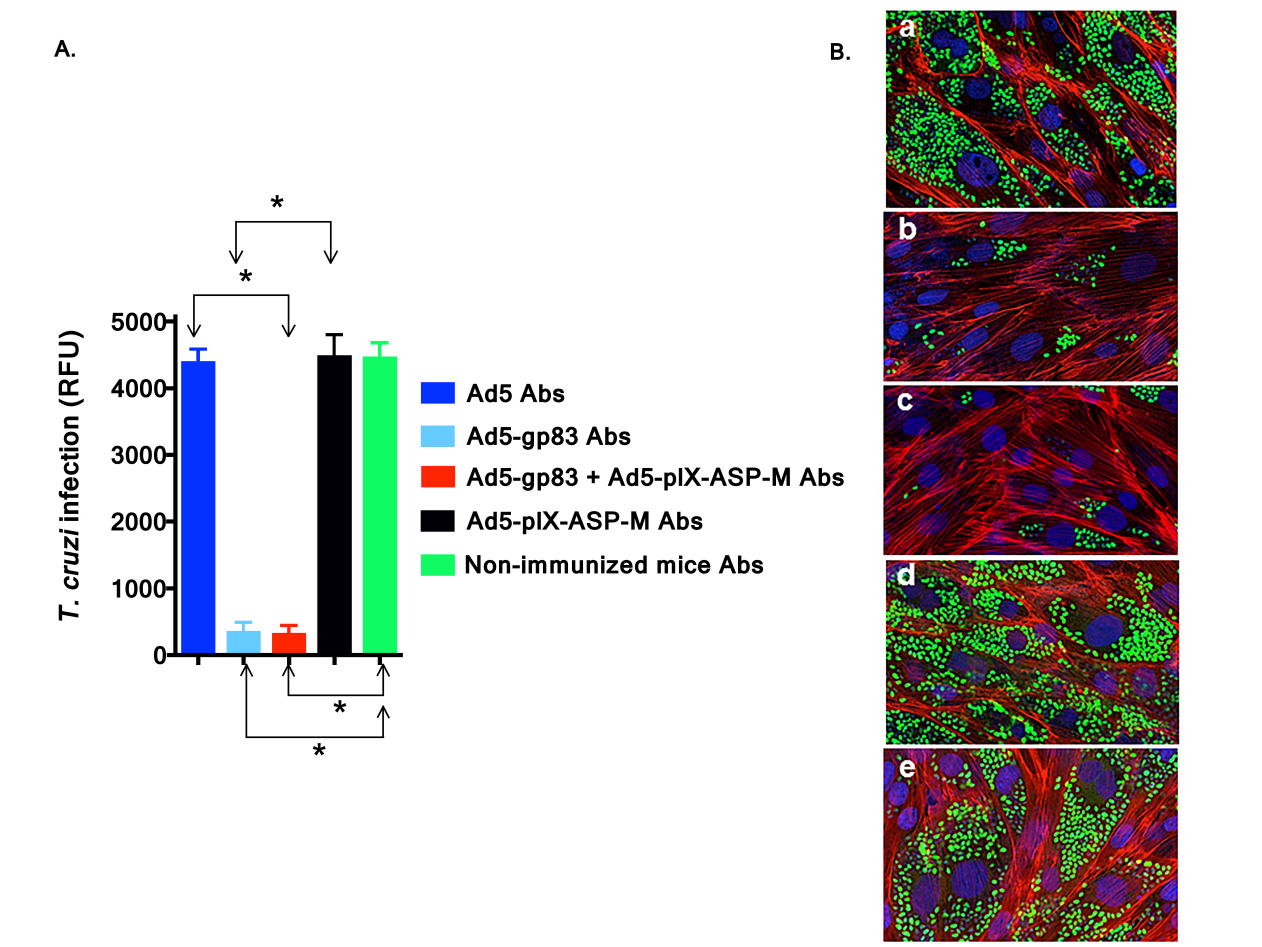
**Mice co-immunized with Ad5-pIX-ASP-M and Ad5-gp83 induce neutralizing antibodies.** (A): Neutralization of *T. cruzi* infection of cardiomyocytes with Abs from vaccinated mice with Ad5, Ad5-gp83, Ad5-gp83 + Ad5pIX-ASP-M, Ad5-pIX-ASP-M, or with Abs from nonimmunized mice. Parasite multiplication within cell monolayers was estimated by determining the fluorescence level of parasites expressing green fluorescence protein, which is indicated as relative fluorescence units (RFU) at 72 h of infection. Data represent the mean values ± SEM of results from triplicate samples. (*) = *P* < 0.0001 by 1-way ANOVA. (B): Fluorescence microscopic observation of the effect of neutralizing antibodies on cardiomyocyte infection by *T. cruzi*. Trypomastigotes expressing GFP were pre-treated with Abs from mice vaccinated with Ad5 (a), Ad5-gp83 (b), Ad5-gp83 + Ad5-pIX-ASP-M (c), Ad5-pIX-ASP-M (d), or with Abs from nonimmunized mice (e) and exposed to cardiomyocytes for 72 h as described in Material and Methods. Abs were obtained from mice injected with either vaccine constructs or with Abs from nonimmunized mice before *T. cruzi* challenge. GFP-expressing amastigotes are seen inside host cells, host cell nuclei are stained blue, and cellular actin filaments are stained red.

Mice that were immunized with Ad5-pIX-ASP-M, Ad5-gp83, or Ad5-gp83 + Ad5-pIX-ASP-M were able to prolong survival after *T. cruzi* challenge compared with mice immunized with Ad5 alone. However, mice co-immunized with a vector containing an amastigote surface epitope (Ad5-pIX-ASP-M) and a vector containing a trypomastigote surface epitope (Ad5-gp83), exhibited a higher survival rate among the immunized groups (Figure 5C). Neutralizing antibodies obtained after immunization from the various groups of mice were able to control or reduce infection of cardiomyocytes by *T. cruzi* as compared with the Ad5-pIX-ASP-M or Ad5 vaccinated or control mice (Figure 6A and 6B). Mice immunization with Ad5-gp83 induces potent neutralizing antibodies (Figure 6), whereas immunization with Ad5-pIX-ASP-M only induced strong CD8^+^ responses (Figure 4C and 4F). However, co-immunizing mice with Ad5-gp83 and Ad5-pIXASP-M induced neutralizing antibodies (induced by Ad5-gp83) (Figure 6) and strong specific CD8^+^ responses induced by Ad5-pIX-ASP-M (Figure 4C and 4F).

## DISCUSSION

A safe and effective vaccine against *T. cruzi* has long been in demand, but has been elusive thus far. Here we demonstrate that the *T. cruzi* epitope capsid-incorporation strategy is a new approach for Chagas vaccine development.

In this study we examine the CD8^+^ responses to the *T. cruzi* amastigote ASP-M epitope that was incorporated into the Ad5 pIX to evaluate whether this vaccine elicits an immune protective response. The insertion of the ASP-M epitope did not affect pIX; major capsid proteins, such as fiber; or the overall fitness of the vector as we previously described for the incorporation of gp83 [22]. The modified vector had normal growth characteristics similar to the wild type Ad5. The insertion of the ASP-M epitope yielded surface exposure as demonstrated by whole-virus ELISA assay. In this study, we also examined the vaccine efficacy of co-immunizing mice with Ad5-pIX-ASP-M carrying the amastigote ASP-M epitope and the Ad5-gp83 carrying the trypomastigote gp83 neutralizing epitope, a vaccine strategy that has not been previously carried out for *T. cruzi.*

Previously, we examined the humoral responses to the *T. cruzi* gp83-epitope that was capsid-incorporated on Ad5 vectors where we generated a recombinant Ad5 vector with an epitope derived from *T. cruzi* [22]. This gp83 epitope was incorporated into the HVR1 region of the major capsid protein hexon. Immunization with the *T. cruzi* capsid-modified vector (Ad5-gp83) elicits a robust neutralizing antibody response and reduces infection in murine experimental models for Chagas disease [22].

In this study we show that co-immunization of mice with the epitope capsid-incorporation strategy of invasive extracellular trypomastigotes and intracellular replicative amastigotes is effective at stimulating *T. cruzi*-specific effector CD8^+^ T-cell responses as well as neutralizing antibodies that protect mice against *T. cruzi* infection by significantly reducing parasitemia and extending survival rates. This co-immunization induced H-2K^k^-restricted cytotoxic and interferon IFNγ producing activated CD8^+^ T-cells, expressing TNFα and neutralizing antibodies. In this co-immunization strategy, the Ad5-pIX-ASP-M component of the vaccine induces intracellular IFNγ expression, which is a marker of CD8^+^ T-cell activation, and CD107a, which is a marker for cytotoxic function, and TNFα. Ad5-gp83, the other component of the vaccine, induces neutralizing antibodies. Thus, we suggest that co-immunizations with Ad5-pIX-ASP-M and Ad5-gp83 with both *T. cruzi* epitopes represent an advancement in the development of a Chagas vaccine.

Our results show that IFNγ and TNFα expression by activated CD8^+^ cells may be required for effective clearance of *T. cruzi*, as demonstrated in transgenic models where lower numbers of IFNγ-producing CD8^+^ cells were unable to block replication of hepatitis B virus [38].

Our results also suggest that ASP-M-activated CD8^+^ T lymphocytes can reduce *T. cruzi* parasitism by secreting IFNγ and TNFα. This may be consistent with studies suggesting that antigen-activated CD8^+^ T lymphocytes can eliminate or control viral infection by secretion of IFNγ and TNFα [38–40].

A major obstacle to using Ad5 for vaccine therapy is that the majority of the population has pre-existing immunity (PEI) resulting from natural exposure to the common cold [41–44]. The antigen capsid-incorporation strategy to some degree can circumvent PEI in mice relative to boost and reboost [22]. One of our future strategies to circumvent PEI is to develop a chimeric Ad5 vector by replacing the entire Ad5 hexon with the hexon from Ad serotype 3 and to develop *T. cruzi* vaccine vectors from rare adenovirus serotypes (e.g., Ad3, Ad35, or Ad36) [45–47].

A protein level similarity search using BLASTP and TBLASTN was conducted against the Uni-Prot database (http://www.uniprot.org) and NCBI RefSeq/genomes database (http://www.ncbi.nlm.nih.gov/refseq). We observed highly significant identity between the peptide epitope of gp83 and the ASP-M epitope to multiple geographically diverse *T. cruzi* strain proteins. Significant protein level similarity to the gp83 epitope was observed among *T. cruzi* strains from Brazil (CL Brener, Y, Sylvio, and Marinkellei), Argentina (CA1), Venezuela (Dm28C), Chile (Tulahuen), Bolivia (SO34 cL4), Colombia (Colombiana), and Mexico (MINOA). The amastigote epitope TEWETGQI presented statistically significant protein identity with proteins from *T. cruzi* strains from Brazil (CL Brener, Y, Brazil, Sylvio, G, Esmeraldo, and Marinkellei), Chile (Tulahuen), Colombia (Colombiana), and Venezuela (Dm28C and JR cl4). Furthermore, both epitopes are present in strains that are drug resistant, partially resistant, and susceptible. Thus, we suggest that both epitopes must be present in the development of molecular vaccines for Brazil, Chile, Venezuela, and Colombia and to cover infections caused by drug-resistant parasites that affect those countries.

We predicted that the ASP-M epitope binds to the HLA-A* 32:05, HLA-A* 32:08, HLA-A* 02:87, HLA-A* 32:06, and HLA-A* 32:20, which are alleles frequent in Hispanic populations where Chagas disease occurs, and to 10 different HLA-A* 02 and 32 alleles. After further genetic analysis (host and parasite), animal experimentation, and pathogen exposure history, there may be a precedent to administer this vaccine of similar vaccines to people in countries exposed to *T. cruzi* strains we identified here.

In summary, we demonstrated that co-immunizations with Ad5-pIX-ASP-M and Ad5-gp83 would be useful in the development of vaccines against Chagas disease, due to the ability of the vector to trigger robust ASP-M-activated CD8^+^ T lymphocytes that reduce *T. cruzi* parasitism by secreting IFNγ and TNFα induced by Ad5-pIX-ASP-M and eliciting neutralizing antibodies via Ad5-gp83 to reduce parasitemia. The antigen capsid-incorporation strategy is attractive for a complex parasite such as *T. cruzi*, because the adenovirus capsid is extremely amenable to the incorporations of multiple linear and discontinuous epitopes of parasite antigens at various life cycles. Therefore, we suggest that this strategy can be manipulated to introduce *T. cruzi* epitopes of immunological importance to induce a robust anti-*T. cruzi* humoral and protective cellular response. Furthermore, our current study can be viewed as a platform to introduce an effective vaccine strategy for other infectious diseases.
